# Patients with chronic lymphocytic leukemia and complex karyotype show an adverse outcome even in absence of *TP53/ATM FISH* deletions

**DOI:** 10.18632/oncotarget.17350

**Published:** 2017-04-21

**Authors:** Anna Puiggros, Rosa Collado, Maria José Calasanz, Margarita Ortega, Neus Ruiz-Xivillé, Alfredo Rivas-Delgado, Elisa Luño, Teresa González, Blanca Navarro, MaDolores García-Malo, Alberto Valiente, José Ángel Hernández, María Teresa Ardanaz, María Ángeles Piñan, María Laura Blanco, María Hernández-Sánchez, Ana Batlle-López, Rocío Salgado, Marta Salido, Ana Ferrer, Pau Abrisqueta, Eva Gimeno, Eugènia Abella, Christelle Ferrá, María José Terol, Francisco Ortuño, Dolors Costa, Carol Moreno, Félix Carbonell, Francesc Bosch, Julio Delgado, Blanca Espinet

**Affiliations:** ^1^ Laboratori de Citogenètica Molecular, Laboratori de Citologia Hematològica, Servei de Patologia i Servei Hematologia, Hospital del Mar, Barcelona, Spain; ^2^ Grup de Recerca Translacional en Neoplàsies Hematològiques, Programa de Recerca en Càncer, Institut Hospital del Mar d’Investigacions Mèdiques (IMIM), Barcelona, Spain; ^3^ Servicio de Hematología, Consorcio Hospital General Universitario, Valencia, Spain; ^4^ Servicio de Citogenética, Departamento de Genética, Universidad de Navarra, Pamplona, Spain; ^5^ Laboratorio de Citogenética y Servicio de Hematología, Hospital Vall d'Hebron, Barcelona, Spain; ^6^ Servei Laboratori Hematologia, ICO-Hospital Germans Trias i Pujol, Institut de Recerca Contra la Leucèmia Josep Carreras (IJC), Universitat Autònoma de Barcelona, Badalona, Spain; ^7^ Secció d’Hematopatologia, Hospital Clínic, Institut d’Investigacions Biomèdiques Augustí Pi i Sunyer (IDIBAPS), Universitat de Barcelona, Barcelona, Spain; ^8^ Servicio de Hematología, Hospital Universitario Central de Asturias, Oviedo, Spain; ^9^ Fundación Pública Galega de Medicina Xenómica, Santiago de Compostela, Spain; ^10^ Servicio de Hematología y Oncología Médica, Hospital Clínico Universitario de Valencia, Valencia, Spain; ^11^ Servicio de Hematología y Oncología Médica, Hospital Universitario Morales Meseguer, Centro Regional de Hemodonación, IMIB-Arrixaca, Murcia, Spain; ^12^ Servicios de Genética y Hematología, Complejo Hospitalario de Navarra, Pamplona, Spain; ^13^ Servicio de Hematología, Hospital Universitario Infanta Leonor, Madrid, Spain; ^14^ Servicio de Hematología, Hospital Txagorritxu, Vitoria, Spain; ^15^ Servicio de Hematología, Hospital de Cruces, Bilbao, Spain; ^16^ Servei d’Hematologia Hospital Universitari de la Santa Creu i Sant Pau, Barcelona, Spain; ^17^ Servicio de Hematología, Hospital Universitario de Salamanca, IBSAL, IBMCC, Centro de Investigación del Cáncer, Universidad de Salamanca, CSIC, Salamanca, Spain; ^18^ Servicio de Hematología, Hospital Universitario Marqués de Valdecilla, Santander, Spain; ^19^ Laboratorio de Citogenética, Servicio de Hematología, Fundación Jiménez Díaz, Madrid, Spain

**Keywords:** CLL, complex karyotype, ATM deletion, TP53 deletion

## Abstract

Genomic complexity identified by chromosome banding analysis (CBA) predicts a worse clinical outcome in CLL patients treated either with standard or new treatments. Herein, we analyzed the clinical impact of complex karyotypes (CK) with or without high-risk FISH deletions (*ATM* and/or *TP53*, HR-FISH) in a cohort of 1045 untreated MBL/CLL patients. In all, 99/1045 (9.5%) patients displayed a CK. Despite *ATM* and *TP53* deletions were more common in CK (25% vs 7%; *P* < 0.001; 40% vs 5%; *P* < 0.001, respectively), only 44% (40/90) patients with *TP53* deletions showed a CK. CK group showed a significant higher two-year cumulative incidence of treatment (48% vs 20%; *P* < 0.001), as well as a shorter overall survival (OS) (79 mo vs not reached; *P* < 0.001). When patients were categorized regarding CK and HR-FISH, those with both characteristics showed the worst median OS (52 mo) being clearly distinct from those non-CK and non-HR-FISH (median not reached), but no significant differences were detected between cases with only CK or HR-FISH. Both CK and *TP53* deletion remained statistically significant in the multivariate analysis for OS. In conclusion, CK group is globally associated with advanced disease and poor prognostic markers. Further investigation in larger cohorts with CK lacking HR-FISH is needed to elucidate which mechanisms underlie the poor outcome of this subgroup.

## INTRODUCTION

Well-established poor prognostic factors in chronic lymphocytic leukemia (CLL) include deletions in 11q, which involve *ATM locus* (del*ATM*), and losses affecting *TP53* gene located in 17p13 (del*TP53*) detected by fluorescence *in situ* hybridization (FISH) [1]. While the adverse prognosis of del*ATM* has been partially overcome by chemoimmunotherapy-based treatments, patients showing *TP53* deletions or mutations are typically resistant to these treatments [2, 3]. New agents acting independently of the p53 pathway have changed the treatment response rate for CLL patients with *TP53* abnormalities [4, 5]. However, recent studies have revealed that current FISH analyses (using Dohner's hierarchical model probes) underestimate the true genomic complexity revealed by chromosome banding analysis (CBA) which predicts a worse clinical outcome in patients treated either with standard treatments or new agents [6–11]. The aims of the present study were: (i) to describe and compare the characteristics and clinical course of CLL patients according to karyotype complexity detected by CBA, (ii) to analyze the impact of complex karyotype (CK) and high-risk FISH deletions (*ATM* and/or *TP53*, HR-FISH) on time to the first therapy and overall survival.

## RESULTS AND DISCUSSION

In the whole series, 435/1043 (41.6%) patients displayed abnormal karyotypes, being 99 (9.5%) complex. While most patients with abnormal but non-CK carried a single aberration (267/336, 80%), cases with CK displayed a median of 4 abnormalities (range: 3-18). Of note, structural aberrations were slightly predominant in both groups (63% and 57% in CK and non-CK, respectively). Notably, abnormal karyotypes from non-CK were mainly restricted to known recurrent CLL aberrations, namely trisomy 12 (37%), 13q14 structural anomalies (17% deletions and 4% translocations), del(11q) (12%), and translocations involving 14q (6%). On the contrary, CK patients displayed a wide variety of abnormalities distributed along the genome, which reflected the genomic instability of this group. Among them, the most frequent were 17p structural rearrangements (deletions, unbalanced translocations or isochromosome 17q) leading to *TP53* loss (26%), trisomy 12 (26%), del(13q) (19%), del(11q) (16%), trisomy 18 (11%) and del(6q) (10%). In addition, clonal evolution by CBA was identified in 33/99 (33%) patients with CK. Detailed karyotypes from those patients from CK group are displayed in Supplementary Table 3.

**Figure 1:**
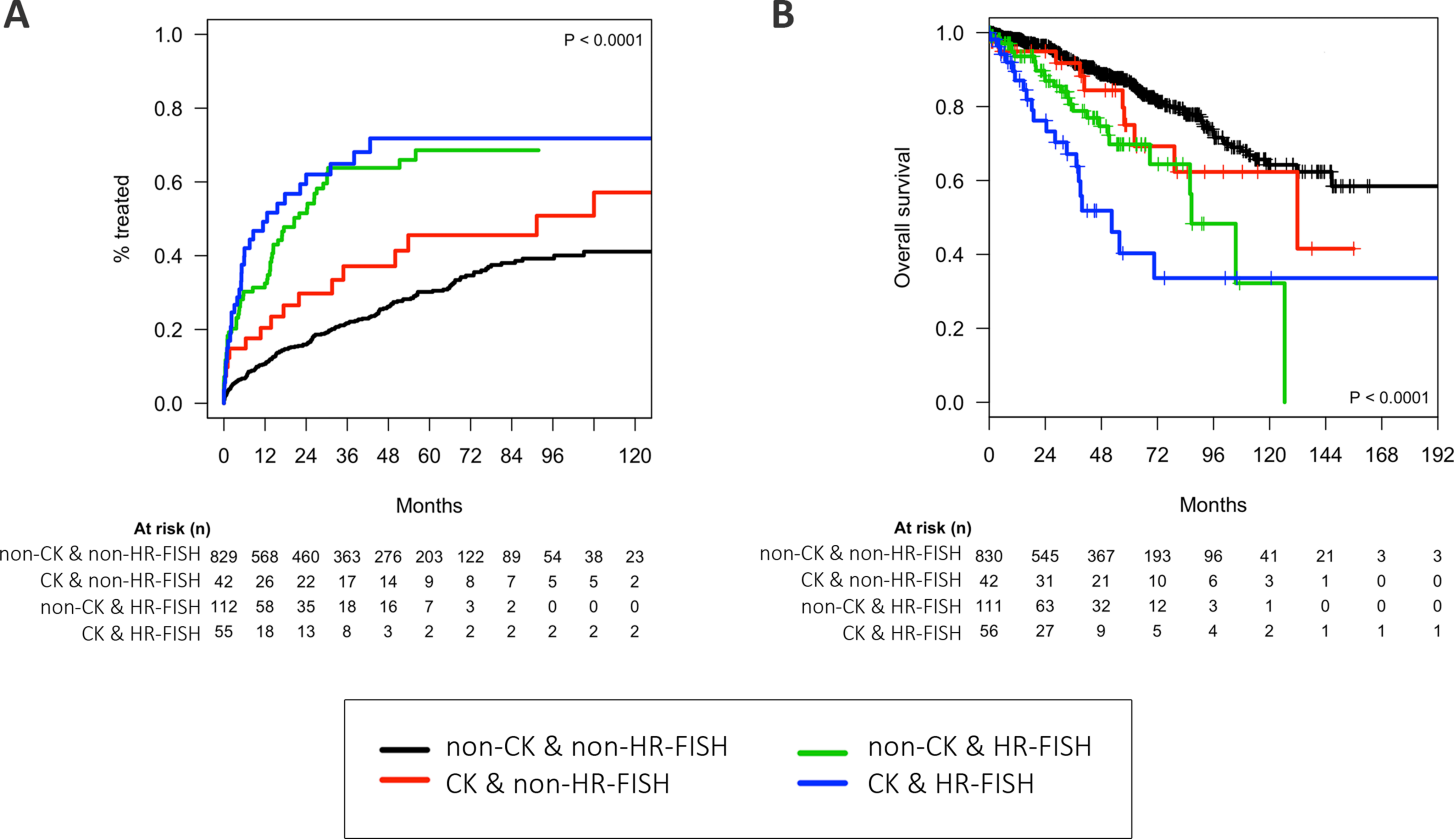
(**A**) Cumulative incidence of treatment (TFT) for the presence of CK and/or HR-FISH and (**B**) Kaplan–Meier plot for overall survival (OS) and the presence of CK and/or HR-FISH.

The CK group included patients with more advanced disease at diagnosis, including a higher proportion of patients diagnosed at Binet stage B/C (22% vs 11%; *P* = 0.001) and higher β2-microglobulin levels (2.6 mg/L vs 2 mg/L; *P <* 0.001). Moreover, features of aggressive disease were also observed: higher CD38 (33% vs 20%; *P* = 0.007) and ZAP-70 (46% vs 30%; *P* = 0.018) positivity rates, del*ATM* (25% vs 7%; *P <* 0.001) and del*TP53* (40% vs 5%; *P <* 0.001). Despite the limited availability of *IGHV* data in this retrospective cohort (*N* = 98/1045), CK group also displayed a significant higher proportion of unmutated *IGHV* genes [71% (15/21) vs 23% (18/77); *P <* 0.001]. Indeed, CK group showed a significant higher two-year cumulative incidence of treatment (48%, 95%CI 36-58%) than non-CK (20%, 95%CI 18-23%; *P <* 0.001), as well as a shorter overall survival (OS) (79 mo vs not reached; *P <* 0.001). The del*TP53* enrichment observed in CK group was in accordance with the previously described frequencies (ranging from 28-50%) and could be underlying genomic instability leading to CK [6, 9, 12–13]. However, only 44.4% (40/90) patients with del*TP53* showed a CK. CK negatively impacted on OS even within del*TP53* cases (38 mo vs 133 mo; *P <* 0.001). In this regard, when patients were categorized regarding both CK and HR-FISH, no relevant differences in clinical characteristics at diagnosis were observed among those patients with at least one high-risk cytogenetic marker (CK and/or HR-FISH) (Table 1). Nevertheless, patients could be stratified in terms of time to first treatment (Figure 1). Of note, while patients with both HR-FISH and CK showed the worst median OS (52 mo) being clearly distinct from those non-CK and non-HR-FISH (median not reached), no significant differences were detected between cases with only CK or HR-FISH (Figure 1). Similarly, if only CK and del*TP53* were considered, no differences in OS for CK patients lacking del*TP53* and those with del*TP53* but non-CK were observed (132 mo vs 89 mo; *P* = 0.269). In the multivariate analysis for OS, both CK and del*TP53* remained statistically significant. Of note, *delTP53* was the predictor showing higher hazard ratio in the multivariate analysis (Table 2). Unfortunately, as this is a retrospective and multicentric study, *TP53* mutational *status* was not routinely assessed and availability of DNA samples was limited. Despite this limitation, *TP53* mutations could be analyzed in 8/42 cases from CK and non-HR-FISH subgroup and all of them were negative. In accordance, although it has been described that nearly 20% of patients with *TP53* abnormalities show mutations in absence of 17p deletion [14, 15], this percentage is much lower in general untreated CLL cohorts (4.5%) [16]. In this regard, despite the limited availability of information regarding *TP53* mutational *status*, only a minority of CK and non-HR-FISH patients could harbor *TP53* mutations and therefore could be misclassified. So that, it could be assumed that other factors than abnormal *TP53* function are underlying the global poor outcome observed in this subgroup. On the other hand, despite the detection of unmutated *IGHV* was significantly associated with shorter OS in the global cohort (55 mo vs 133 mo; *P <* 0.001), the limited availability of *IGHV* data did not allow the stratification by both *IGHV* and CK in the OS assessment.

**Table 1:** Baseline characteristics of patients with unfavourable cytogenetic markers (CK and/or HR-FISH) at diagnosis

Patients characteristics	CK and non-HR-FISH (*N* = 42)	non-CK and HR-FISH (*N* = 113)	CK and HR-FISH (*N* = 57)	*P*-value
**Median age at diagnosis (range)**	73 (46–87)	67 (27–90)	69 (45–88)	0.322
**Male**	31 (73.9%)	78 (69.0%)	35 (61.4%)	0.284
**Diagnosis**				
** MBL**	10 (23.8%)	13 (11.5)	4 (7.0%)	0.039
** CLL**	32 (76.2%)	100 (88.5%)	53 (93.0%)	
**Advanced Binet stage** *	9/42 (21.4%)	33/109 (30.3%)	11/56 (19.6%)	0.262
**Lymphadenopathy**	16/42 (38.1%)	56/110 (50.1%)	28/55 (50.9%)	0.323
**Splenomegaly**	3/40 (7.5%)	15/109 (13.8%)	8/53 (15.1%)	0.512
**Hepatomegaly**	2/42 (4.8%)	11/112 (9.8%)	2/56 (3.6%)	0.266
**Absolute white blood cell count (x10^9^/L)**	16 (6–164)	17 (4–168)	21 (5–89)	0.122
**Absolut lymphocyte count (x10^9^/L)**	11 (2–105)	11 (1–155)	14 (2–82)	0.091
**Hemoglobin (g/dL)**	14 (9–17)	14 (6–18)	14 (8–17)	0.219
**Platelets (x10^9^/L)**	190 (42–473)	191 (10–413)	173 (34–331)	0.470
**Lactate dehydrogenase (IU/L)**	332 (160–1279)	340 (139–1094)	383 (156–1082)	0.237
**Beta-2 Microglobulin (mg/L)**	2.4 (1.0–10.0)	2.2 (1.0–13.4)	2.6 (1.1–8.4)	0.305
ZAP-70 positive**	7/20 (35.0%)	17/62 (27.4%)	16/31 (51.6%)	0.071
CD38 positive*	11/31 (35.5%)	33/96 (34.4%)	14/42 (33.3%)	0.982
**Unmutated** ***IGHV***	1/5 (20.0%)	2/2 (100%)	13/14 (92.9%)	0.003
**FISH**				
** 13q deletion**	16/42 (38.1%)	54/110 (49.1%)	34/55 (61.8%)	0.064
** Trisomy 12**	24/42 (57.1%)	15/109 (13.8%)	7/54 (13.02%)	< 0.001
** 11q deletion (*ATM***)	–	68/113 (60.2%)	25/57 (43.9%)	N.A.
** 17p deletion (*TP53***)	–	50/113 (44.2%)	40/57 (70.2%)	N.A.

Values are given as median (range) or number (%). Hemoglobin is expressed as mean (range). N.A. Not applicable.

*Binet stage was not available in five patients (information regarding physical examination and/or analytical parameters not provided).

**Positivity was considered when ZAP-70 > 20% and CD38 > 30%.

**Table 2:** Univariate and multivariate analysis on median overall survival (OS)

Variable		Univariate		Multivariate	
		Median OS in months (95% CI)	*P*-value	Hazard ratio (95% CI)	*P*-value
**Complex karyotype (CK)**	Positive	79 (23–135)	< 0.001	1.66 (1.06–2.59)	0.027
	Negative	NR			
**ATM deletion**	Positive	87 (64–108)	0.003	1.51 (0.93–2.43)	0.093
	Negative	NR			
**TP53 deletion**	Positive	56 (34–77)	< 0.001	3.03 (1.93–4.77)	< 0.001
	Negative	197 (107–287)			

NR: Not reached.

Mounting evidence suggests a relevant role of CBA in risk assessment of CLL patients. Indeed, Rigolin *et al*. demonstrated that 25-37% of patients showing no aberrations by FISH carried chromosomal abnormalities not covered by the standard FISH panel, which strongly correlated with worse prognosis [17]. Moreover, Jaglowski *et al*. described that both the number of karyotypic abnormalities as a continuous variable and the CK detection (defined as ≥ 5 abnormalities) could predict a worse clinical outcome in patients undergoing reduced-intensity conditioning allogeneic stem cell transplantation; remarkably, both variables retained their predictive prognostic value even within patients with HR-FISH [6]. In this regard, a recent study described the impact of CK as an independent unfavorable prognostic factor in a prospective trial using chlorambucil-based regimens in CLL patients with relevant comorbidity. In accordance to our results, an important proportion of patients with CK lacking *TP53* abnormalities (73% cases with CK) showed a dismal survival, equivalent to those patients with sole *TP53* lesions [11]. Novel evidences suggesting the value of CBA have arisen with the recent development of new treatment agents in CLL. Initially, Thompson *et al*. demonstrated that CK showed stronger impact on outcome than del*TP53* in CLL patients treated with ibrutinib [10]. Furthermore, this negative impact on the outcome of ibrutinib-treated patients has been subsequently confirmed after a longer 5-year follow-up [18], as well as in a large “real-world” multicentric analysis of patients treated with novel agents in CLL [19]. It is noteworthy that a lack of any adverse prognostic effect of CK on idelalisib-treated have been recently reported [20]. However, this finding should be confirmed in a larger cohort of CLL undergoing idelalisib-based regimens. All these findings point out CK as a potent prognostic factor in the new therapies era, especially considering that ibrutinib treatment increased survival in treatment-naïve older CLL patients without *TP53* deletion irrespectively of other historical poor prognostic factors, such as del*ATM* or unmutated *IGHV* [5]. Nevertheless, patients with CK are indeed a heterogeneous group: patients harboring multiple trisomies, namely +12+18+19, and non-HR-FISH show a particularly benign clinical evolution [21]. Herein, we detected nine such cases presenting a better outcome compared with the remaining CK cases (data not shown). This observation suggests that criteria for CK definition in CLL should be revised; and that cytogenetic complexity defined by solely numerical aberrations should not be unquestionably considered as an unfavorable prognostic marker in this entity [21]. Even though previous publications evaluate cytogenetic complexity by the number of abnormalities detected by CBA, as well as the number or total size of altered regions by genomic microarrays, standard criteria focusing on both the number and the nature of the abnormalities are highly needed to provide a more accurate prognostic marker in CLL.

In conclusion, we report the existence of a subgroup of CLL patients with CK lacking HR-FISH abnormalities (del*ATM*/del*TP53*) that show an equivalent impaired clinical evolution as those with HR-FISH and non-CK. Despite CK group is globally associated with advanced disease and poor prognostic markers, genetic basis other than *TP53/ATM* dysfunction underlying the heterogeneity of CK remain to be elucidated. In order to better determine the patients’ prognosis according to genomic complexity detected by CBA, and make possible to select tailored therapies, further investigation in larger patient cohorts with CBA data is required.

## MATERIALS AND METHODS

### Patient selection

We selected 1045 CLL and monoclonal B-cell lymphocytosis (MBL) patients (821 and 224, respectively) with available simultaneous karyotype and FISH at diagnosis or prior to therapy from the CLL/MBL database of the Grupo Cooperativo Español de Citogenética Hematológica (GCECGH) and Grupo Español de Leucemia Linfática Crónica (GELLC). Of note, MBL individuals were also included, as MBL represents an initial phase in the disease evolution, some of them harbor poor prognosis cytogenetics and could evolve to CLL requiring treatment during follow-up. The median time from diagnosis to CBA was 0.5 months (range, 0-229). The study was approved by the ethical committee of Hospital del Mar, Barcelona (ref. 2016/6861/I).

Clinical information collected at diagnosis included demographics (age and gender), Binet stage, physical examination, and analytical information regarding absolute white blood cell and lymphocyte counts, platelet count, hemoglobin level, as well as lactate dehydrogenase (LDH) and serum beta2-microglobulin (B2M) concentrations. When available, CD38 and ZAP-70 expression and mutational *status* of immunoglobulin heavy chain genes (*IGHV*) were also compiled. Evolutive data on treatment administration and last follow-up were recorded (Supplementary Table 1). Treatment administration and response evaluation were performed according to current guidelines [22–24]. At the end of the study, 30.2% of patients had required therapy. The most frequently used therapies in first-line treatments are detailed in Supplementary Table 2. Of note, only four of the included patients received novel agents acting independently of *TP53* pathway (ibrutinib) during follow-up.

### Chromosome banding analyses and fluorescence *in situ* hybridization

Chromosome banding analyses were mainly assessed on peripheral blood samples (*n* = 881, 84%), whereas bone marrow and lymph node samples were only analyzed in 159 and five cases, respectively. Cell cultures were set for 72 hours with TPA (12-O-tetradecanoyl-phorbol-13-acetate) according to standard procedures. A minimum of 20 metaphases were analyzed when possible. Karyotypes were described following the International System for Human Cytogenetic Nomenclature (2013) [25]. Only those abnormalities detected in at least two metaphases or those detected in a single metaphase but further confirmed by FISH or in a subsequent CBA were considered. CK was defined as the presence of three or more chromosomal abnormalities in a single clone. Data from routine FISH panel for *TP53* (17p13), *ATM* (11q22), D13S319 (13q14) and chromosome 12 centromeric region were obtained simultaneously to CBA.

### Statistical analysis

Firstly, patients were classified according to the presence of CK independently of FISH results (CK vs non-CK). Discrete variables were compared between groups by Chi-square or Fisher exact tests, while Mann-Whitney test was used for continuous variables. Time to first treatment (TFT) was defined as time from sampling to start of treatment or last follow-up; overall survival (OS) was defined as time from sampling to death or last follow-up. Both TFT and OS were assessed using cumulative incidence and Kaplan–Meier plots, respectively and the effect of different covariates was evaluated using the Gray's and log-rank test, respectively. The maintenance of the independent predictive value on OS was assessed in multivariate analyses using Cox proportional hazards regression models. To further clarify the impact of high-risk FISH deletions (*ATM* and/or *TP53*, HR-FISH) in CK prognosis, a second classification including CK and HR-FISH was performed. TFT and OS from four groups were compared: (1) CK and HR-FISH; (2) CK and non-HR-FISH; (3) non-CK and HR-FISH; and (4) non-CK and non-HR-FISH. Statistical analyses were performed using SPSS v.22 software (SPSS Inc, Chicago, IL, USA) and R v.3.2.2. Results were considered statistically significant for *P*-values < 0.05.

## SUPPLEMENTARY MATERIALS TABLES




