# Supplementary material for: Patients with chronic lymphocytic leukemia and complex karyotype show an adverse outcome even in absence of TP53/ATM FISH deletions
Source: Oncotarget. 2017 Apr 21;8(33):54297–303. doi: 10.18632/oncotarget.17350 (PMC5589581; doi:10.18632/oncotarget.17350)
Supplement: Supplementary file 1 [file oncotarget-08-54297-s001.pdf]

Patients with chronic lymphocytic leukemia and complex karyotype show an adverse outcome even in absence of *TP53/ATM* FISH deletions

Supplementary Materials

Supplementary Table 1: Baseline characteristics of 1045 CLL and MBL patients at diagnosis and last follow-up

Patients characteristics	
Median age at diagnosis (range)	68 (27–94)
Male	632 (60.5%)
Diagnosis	
MBL	224 (21.4%)
CLL	821 (78.6%)
Binet stage (*n* = 802)	
A	685 (85.4%)
B	89 (11.1%)
C	28 (3.5%)
Lympadenopathy (*n* = 1021)	316 (31.0%)
Splenomegaly (*n* = 1014)	65 (6.4%)
Hepatomegaly (*n* =1032)	38 (3.7%)
Absolute white blood cell count ($\times 10^9$/L)	15 (3–177)
Absolut lymphocyte count ($\times 10^9$/L)	10 (1–170)
Hemoglobin (g/dL)	14 (5–18)
Platelets ($\times 10^9$/L)	195 (10–555)
Lactate dehydrogenase (IU/L)	310 (81–1548)
Beta-2 Microglobulin (mg/L)	2.1 (1.0–19.0)
ZAP-70 positive (*n* = 501)*	170 (31.3%)
CD38 positive (*n* = 836)*	174 (20.8%)
Unmutated *IGHV* (*n* = 98)	33 (33.7%)
FISH	
13q deletion	475 (45.4%)
Trisomy 12	190 (18.2%)
11q deletion (*ATM*)	93 (8.9%)
17p deletion (*TP53*)	90 (8.6%)
Median follow-up (months)	46 (0–272)
Therapy during follow-up	316 (30.2%)
Number of treatments	1 (1–8)
Died during follow-up	167 (16.0%)

*Positivity was considered when ZAP-70 > 20% and CD38 > 30%.Values are given as median (range) or number (%). Hemoglobin is expressed as mean (range).

Supplementary Table 2: Most frequently administered therapies in first-line treatment

Therapy	Frequency
**Chlorambucil**	31.9%
**Rituximab + fludarabine + cyclophosphamide**	18.3%
**Fludarabine + cyclophosphamide (±mitoxantrone)**	8.7%
**Fludarabine**	4.6%
**R-CHOP**	3.2%
**Bendamustine + Rituximab**	2.3%

R-CHOP: *rituximab + cyclophosphamide + Adriamycin + vincristine + prednisone*.

Supplementary Table 3: Karyotype result and *ATM/TP53 status* from the 99 patients with CK.
See Supplementary_Table_3
